# Multiple motives for cannabis use: identifying common motive combinations and exploring differences across combinations

**DOI:** 10.1186/s42238-025-00362-z

**Published:** 2025-11-21

**Authors:** Christina Dyar, Julia Curtis, Christine M. Lee

**Affiliations:** 1 College of Nursing, Ohio State University, Columbus, United States; 2 Department of Psychiatry and Behavioral Sciences, University of Washington School of Medicine, Seattle, USA

**Keywords:** Cannabis, Motives, Ecological momentary assessment

## Abstract

**Background:**

Several ecological momentary assessment studies have examined event-level associations between motives for cannabis use and cannabis use outcomes (e.g., duration of intoxication, consequences), and endorsing multiple motives during the same observation has been linked to heavier cannabis use and more consequences. However, it remains unclear whether specific combinations of motives are differentially associated with cannabis use outcomes above and beyond the effect of endorsing more motives.

**Methods:**

We utilized ecological momentary assessment data from 571 young adult females who regularly used cannabis to conduct a multilevel latent class analysis of observations during which multiple motives for cannabis use were endorsed and compared results to those derived from a count of the number of motives endorsed.

**Results:**

Endorsing more motives (operationalized as a count of motives) was associated with heavier cannabis use and more consequences of use at the event-level. Six common combinations of motives (i.e., classes) were identified in the latent class analysis of observations with multiple motives. Results indicated differences among motive classes with the same number of motives. For example, the two classes characterized by the endorsement of three motives displayed different patterns with one (*coping*,* social*,* enhancement*) being consistently associated with heavier use than most classes characterized by endorsing two motives, while the other (*coping*,* enhancement*,* medical*) was not consistently associated with heavier use. Further, one class characterized by endorsement of two motives (*coping and medical*) was consistently associated with lighter use and fewer consequences compared to other classes characterized by endorsement of two motives.

**Conclusion:**

Results indicate that specific combinations of motives are associated with differences in cannabis use outcomes above and beyond the effect of simply endorsing more motives.

## Introduction

Ecological momentary assessment studies (EMA; multiple surveys per day) examining event-level associations between motives for cannabis use and cannabis use outcomes (e.g., number of sessions of cannabis use, duration of intoxication, subjective intoxication, consequences) have produced mixed evidence (Bonar et al. 2017; Dyar and Feinstein 2025; Jackson et al. 2021; Pearson et al. 2020). However, these studies have rarely considered the effect of endorsing multiple motives for cannabis use during the same observation, despite the high frequency with which multiple motives are endorsed (Stevens et al. 2021). A recent study linked the endorsement of more motives for cannabis use with heavier use and more consequences at the event-level, but it remains unclear whether certain combinations of motives may be more strongly associated with cannabis use outcomes than others (Stevens et al. 2021). The current study utilized a multilevel latent class analysis to identify motives that are commonly endorsed together and examined differences in cannabis use outcomes based on the combination of motives endorsed. The results of this more nuanced approach were then compared to the results of associations between a count of the number of motives endorsed and cannabis use outcomes to determine what is gained by using a latent class approach to understanding multiple motive endorsement. We utilized a sample of young adult females who used cannabis regularly which included an oversampling of sexual minority cisgender women (e.g., lesbian, bisexual, and queer women) and gender diverse individuals assigned female at birth (i.e., individuals who identify outside of the gender binary; e.g., nonbinary, genderqueer), who are at elevated risk for heavy cannabis use and cannabis use disorder (Krueger et al. 2020; Watson et al. 2020).

### Event-level associations between motives and cannabis use outcomes

EMA studies examining associations between endorsing specific motives and cannabis use outcomes have produced mixed results, with findings varying across outcomes. For example, studies have linked enhancement motives (i.e., using cannabis to enhance positive experiences) with more sessions of cannabis use and consequences consistently, but results have been more mixed for other outcomes (Dyar and Feinstein 2025; Jackson et al. 2021; Pearson et al. 2020). Two studies have linked enhancement motives with higher subjective intoxication (Dyar and Feinstein 2025; Jackson et al. 2021) but another did not (Pearson et al. 2020), and results are similarly mixed for quantity of cannabis consumed (Bonar et al. 2017; Pearson et al. 2020). In a similar pattern, social motives (i.e., using cannabis to enhance a social situation) have been linked to more sessions of cannabis use (Dyar and Feinstein 2025; Jackson et al. 2021), subjective intoxication (Dyar and Feinstein 2025; Jackson et al. 2021), quantity consumed (Bonar et al. 2017), and consequences (Pearson et al. 2020), but at least one study has failed to find a significant association for each of these effects (Bonar et al. 2017; Dyar and Feinstein 2025; Jackson et al. 2021; Pearson et al. 2020). The same pattern holds for associations between coping motives (i.e., using cannabis to reduce negative emotions) and cannabis use outcomes (Bonar et al. 2017; Dyar and Feinstein 2025; Jackson et al. 2021; Pearson et al. 2020). One potential reason for these mixed findings may be the focus of prior studies on the unique effects of endorsing specific motives over and above other motives, which does not directly consider the effect of endorsing multiple motives.

Fewer studies have examined other motives at the event-level. Conformity motives (i.e., using cannabis because others are using cannabis or to fit in) have been linked with more sessions and consequences (Pearson et al. 2020) but not with quantity or subjective intoxication (Bonar et al. 2017; Pearson et al. 2020), while expansion motives (i.e., using to expand awareness or alter one’s perspective) have been linked with higher subjective intoxication, but not with other outcomes (Bonar et al. 2017; Pearson et al. 2020). We are aware of only one study to examine sleep motives for cannabis use (i.e., to help one sleep) at the event-level, which found no association between sleep motives and consequences (Goodhines et al. 2019). Further, we are not aware of any EMA studies examining medical motives for use (e.g., to reduce pain or nausea) at the event-level. Given limited EMA research on several common motives for cannabis use and mixed evidence for associations between enhancement, social, and coping motives and cannabis use outcomes, further research on EMA motives for use is needed.

### Endorsing multiple motives for cannabis use

Few studies have examined endorsing multiple motives for cannabis and most have focused on the individual-level. Cross-sectional studies have linked strong endorsement of multiple motives with more frequent cannabis use and more consequences of cannabis use (Amiet et al. 2020; Cloutier et al. 2019), but we are aware of only one study to examine these associations at the event-level. Stevens et al. (2021) demonstrated that endorsing multiple motives on cannabis use days is common, with an average of 1.5 motives endorsed. Further, they found that endorsing more motives was associated with more cannabis use sessions and consequences of use. The authors used a count of the number of motives endorsed to operationalize endorsement of multiple motives. While this approach is parsimonious, it does not allow for the examination of differences based on the specific combination of motives endorsed. Rather, this approach assumes that observations in which the same number of motives are endorsed result in the same cannabis use outcomes. Latent class analyses (focused on the observation level in a multilevel model), which identify groups of observations based on shared characteristics, would allow for the identification of distinct sets of observations based on specific combinations of motives endorsed. This approach would identify common motive combinations and allow for comparisons across observations during which multiple motives were endorsed based on the specific combination of motives.

### The current study

The current study aimed to advance our understanding of associations between motives and cannabis use outcomes at the event-level. Specifically, we aimed to compare existing statistical methods for examining event-level associations between motives for cannabis use and cannabis use outcomes (i.e., unique effects of each motive above and beyond other motives; and a count of the number of motives endorsed on a specific day) to a novel approach for examining these associations (i.e., latent class analysis of observations with multiple motive endorsement to identify commonly used combinations of motives and whether they are differentially associated with cannabis use outcomes). The ultimate goals of these analyses were to determine what each approach can tell us about associations between motives and cannabis use outcomes. We tested hypotheses among a sample of young adult females who used cannabis regularly, which included an oversampling of sexual minority cisgender women and gender diverse individuals assigned female at birth, who are at elevated risk for heavy cannabis use and cannabis use disorder (Krueger et al. 2020).

First, we aimed to present event-level associations between motives for cannabis use and cannabis use outcomes, with all motives entered as simultaneous predictors, consistent with the majority of prior research in this area. Based on prior studies, we hypothesized that enhancement, social, and coping motives would be associated with more sessions of cannabis use, longer intoxication, higher subjective intoxication, and more consequences at the event-level. Associations for other motives (conformity, expansion, sleep, and medical) were not specified given limited prior research.

Second, we aimed to examine associations between the number of motives endorsed and cannabis use outcomes, consistent with the approach used by Stevens et al. (2021). We hypothesized that endorsing more motives would be associated with more sessions of cannabis use, longer intoxication, higher subjective intoxication, and more consequences at the event-level. We also aimed to explore whether specific motives were associated with the number of additional motives endorsed during the same observation (e.g., are endorsing coping motives associated with endorsing more or fewer other motives).

Third, we aimed to conduct a multilevel latent class analysis of observations during which multiple motives for cannabis use were endorsed. The goal of this was to identify common combinations of motives that are endorsed together and examine differences across observations with multiple motives based on the combination of motives endorsed. As latent class analyses are data driven and there is a lack of prior research using this approach at the event-level, we did not hypothesize which combinations of motives would be derived from the analysis. Further, given that we did not hypothesize which classes would be derived from the latent class analysis, we also did not provide hypotheses for differences across classes on cannabis use outcomes.

## Methods

### Participants and procedures

The current analyses used data from a longitudinal study of substance use among a sample of young adult females, including sexual minority and heterosexual cisgender women and gender diverse sexual minorities assigned female at birth. Participants were recruited via online advertisements on social media (e.g., Facebook) between January and December 2024. The study included a baseline assessment (day 0) and a 14-day EMA study (days 1–14). During the EMA period, participants completed one survey in the morning (8:00am-1:00pm in their time zone) and one in the evening (6:00pm-12:00am in their time zone). The study received IRB approval at Ohio State University.

Eligible participants were U.S. residents; age 18–25; were assigned female at birth, identified as (a) a sexual minority and a woman, (b) a sexual minority and gender diverse (e.g., nonbinary, genderqueer), or (c) heterosexual and a woman; and used cannabis at least four times in the past month at baseline.^1^ Participants were paid up to $140: $30 for baseline, $2.50 for each EMA survey, and $10 bonus for each 6 surveys completed in a row. Participants who screened eligible based on their eligibility survey were sent a text message by study team members to verify eligibility. Participants were asked to text demographic information (i.e., age, state of residence, email address), which was cross-checked with their responses in the eligibility survey. Any participants who provided incorrect information were excluded from the study. Participant information (e.g., email, phone number, names) was also cross-checked to identify duplicates and prevent participants from being enrolled in the study multiple times. This study was not pre-registered and the set of analyses presented in the manuscript was a secondary analyses (i.e., not a direct test of a study aim).

A total of 653 participants completed the baseline assessment. The analytic sample included all individuals who reported cannabis use at least once during the EMA (*n* = 571). The sample was diverse in race/ethnicity, with 49.4% identifying with at least one marginalized racial/ethnic identity. Participants included 128 heterosexual cisgender women (22.4%), 291 sexual minority cisgender women (50.9%), and 152 gender diverse sexual minorities (26.6%). See Table 1 for more detailed demographic information.

**Table 1.** Demographics of analytic sample at baseline (N = 571)

Demographic Variable	*n*	%
Sexual Identity		
Lesbian/Gay	170	29.8%
Bisexual	139	24.3%
Pansexual	34	6.0%
Queer	100	17.5%
Heterosexual	128	22.4%
Race/Ethnicity^a^		
White	367	64.3%
Black	108	18.9%
Latine	84	14.7%
Asian	83	14.5%
Other Race/Ethnicity	30	5.3%
Gender Identity		
Cisgender Woman	419	73.4%
Gender Minority	152	26.6%
Age (*M*,* SD*)	22.67 (1.98)	

^a^Percentages add up to more than 100% because participants could select multiple racial/ethnic identities

## Measures

### EMA measures

#### Cannabis use motives

Participants were asked whether they used cannabis to cope (3 items; e.g., “to reduce my anxiety”); to enhance positive emotions (1 item; “to feel good or have fun”); to enhance social situations (1 item; “to be social or make a social gathering more fun”); to conform (1 item; “because others were using marijuana); to expand one’s perspective (1 item; “to alter my perspective or think differently”); to treat pain or nausea (“to relieve pain or nausea”); or to sleep (1 item; “to help me sleep”). Response options were no (0) and yes (1). Participants could endorse multiple motives. Items were adapted from Patrick et al. (2019). Consistent with Dyar et al. (2022), the three coping motive items were used to create a binary variable (0 = endorsement of no coping motives, 1 = endorsement of at least one coping motive).

#### Cannabis use outcomes

 The following items were assessed when participants indicated having used cannabis. Frequency of cannabis use, duration of intoxication, and subjective intoxication were adapted from the Cannabis Use Inventory, and participants were asked to answer these questions thinking about their use since the last survey (Cuttler and Spradlin 2017).

##### Frequency of cannabis use

Was assessed by the item “How many separate occasions/sessions of marijuana use did you have?” Response options ranged from 0 to 50+.

##### Duration of intoxication

Was measured by asking “How many hours were you high?” Responses were provided in increments of one hour from 0 to 12 + hours.

##### Subjective intoxication

 Was assessed by asking “How high did you get when you used marijuana?” on a scale of 0 (not at all high) to 4 (extremely high).

##### Cannabis consequences

Were measured only during morning assessments (and asked about the past 24 h) by using eight items from two measures of marijuana consequences (Lee et al. 2021; Simons et al. 2012). Participants were asked which “of the following things happened to you as a result of your marijuana use yesterday?” (e.g., “I felt dizzy or sick”). A sum of consequences was calculated.

### Analytic plan

Analyses were conducted in Mplus 8.11. There were 5,321 completed surveys from 571 participants in which cannabis use was reported (42% of all observations). The median individual-level completion rate was 89.3% (*M* = 76.7%, *SD* = 28.0%). Within completed surveys, < 1% of data were missing and were handled using Bayesian methods in Bayesian analyses (Asparouhov and Muthén 2010) and full information maximum likelihood in analyses using maximum likelihood estimation (i.e., latent class analyses). Only observations in which cannabis use was reported were included in analyses as cannabis use motives were only assessed during these observations.

Bayesian multilevel structural equation modeling (MSEM) with non-informative priors was used for multilevel analyses. MSEM utilizes latent variables, rather than group- and grand-mean centering, to separate within- from between-person variance (Lüdtke et al. 2008). By removing the between-person variance from the event-level variance, the event-level variables indicate the extent to which an individual was experiencing more/less of a construct than usual (above/below their person mean) on a particular day (e.g., experiencing shorter/longer intoxication than usual). We used Markov Chain Monte Carlo (MCMC) algorithms to generate a series of 10,000 random draws from the multivariate posterior distribution of our sample for each model. Trace plots and the Gelman-Rubin potential scaling reduction (PSR) were used to determine whether convergence was achieved (Depaoli and Clifton 2015; Muthén 2010).

In the first set of models (see study aim 1), we examined within-person (i.e., event-level) and between-person associations between all motives entered as simultaneous predictors and cannabis use outcomes (i.e., number of sessions, duration of intoxication, subjective intoxication, and consequences). Within-person associations between motives and cannabis use outcomes were allowed to vary across individuals. In all models, we controlled for day of assessment (e.g., day 1- day 14) and assessment type (weekend/weekday; morning/evening) at the within-person level. Age, sexual identity, gender identity, and race/ethnicity were included as covariates at the between-person level.

In the second set of models (see study aim 2), we examined within-person and between-person associations between specific motives and the number of cannabis use motives endorsed as well as associations between the number of motives endorsed and cannabis use outcomes (i.e., number of sessions, duration of intoxication, subjective intoxication, and consequences). In models involving associations between specific motives and number of additional motives endorsed, the specific motive that acted as the predictor was excluded from the number of motives variable so that the outcome reflected the number of additional motives reported during observations when a specific motive was endorsed. Within-person associations between specific motives, number of motives endorsed, and cannabis use outcomes were allowed to vary across individuals.

In the third set of models (see study aim 3), we conducted a multilevel latent class analyses (LCA) in which observations were classified into groups based on the motives endorsed. As Bayesian modeling is not available for these models in Mplus, we utilized maximum likelihood estimation. Given that this analysis aimed to understand differences across observations in which multiple motives were reported, only observations during which multiple cannabis use motives were reported were included (2,955 observations from 498 participants). All eight motives were included in the first LCA, but expansion and conformity did not help to differentiate classes and were excluded from the final LCA models. Models with 2 to 8 latent classes at the within-person level were estimated. Bayesian Information Criterion (BIC), sample size-adjusted BIC, Vuong Lo-Mendell-Rubin (VLMR) likelihood ratio tests, parametric bootstrapped likelihood ratio tests (BLRT), entropy, smallest class size, and class interpretability were used to select the number of classes (Dziak et al. 2014; Nylund et al. 2007). Lower BIC values indicate the preferred model, and significant VLMR or BLRT indicate a preference for the current model. Higher entropy indicates more certainty about the assignment of observations to latent classes, so models with higher entropy scores are preferred. We considered size of the smallest profiles because small classes (< 1% of observations) may indicate over-extraction (Hipp and Bauer 2006).

Next, associations between latent class membership and cannabis use outcomes were examined. While two and three-step approaches are preferred for latent class analyses in single level models (Asparouhov and Muthén 2014), there are currently no statistical programs that implement two or three step approaches in multilevel models that could accommodate our final LCA model. The latent class solution changed when cannabis use outcomes were included in a single step LCA analysis (i.e., when both motives and cannabis use outcomes were included as indicators of latent classes). This is common in single-step LCA analyses and indicates that the composition of the latent classes are changing to produce stronger associations between the latent classes and the outcome, introducing bias (Bakk and Kuha 2018). Therefore, we treated the most likely class membership variable as an observed variable and conducted follow-up Bayesian MSEM. While this approach does not take the uncertainty of class assignment into account, this was the only feasible statistical option at this time due to the lack of available two and three step approaches for multilevel LCA models that could accommodate our final LCA. We utilized most likely latent class membership as a within-person predictor of cannabis use outcomes and estimated random slopes for associations between each class and the cannabis use outcome. Pairwise comparisons for all pairs of latent classes were calculated and a correction for multiple comparisons was conducted (i.e., the Benjamini-Hochberg correction; Ferreira and Zwinderman 2006).

## Results

The most commonly endorsed motives were coping (79% of cannabis use observations), followed by enhancement (40%), sleep (25%), medical (18%), social (13%), expansion (11%), and conformity (7%). On average, participants endorsed 1.93 motives during cannabis use observations. See Table 2 for additional means, standard deviations, and intraclass correlations.

**Table 2.** Means, variances, and intraclass correlations

	Mean/Proportion Endorsed	Standard Deviation	Range	Intraclass Correlation
Coping Motives	0.79	-	0–1	0.23
Social Motives	0.13	-	0–1	0.18
Enhancement Motives	0.40	-	0–1	0.18
Sleep Motives	0.25	-	0–1	0.29
Medical Motives	0.18	-	0–1	0.44
Altered Perspectives Motives	0.11	-	0–1	0.28
Conformity Motives	0.07	-	0–1	0.29
Number of Motives Endorsed	1.93	0.70	0–7	0.43
Number of Sessions	1.71	1.10	0–30	0.50
Duration Intoxication	3.01	1.25	0–12	0.43
Subjective Intoxication	1.81	0.48	0–4	0.34
Consequences	0.51	0.89	0–7	0.44

The proportion of observations during which each motive was endorsed are presented for binary motives variable, while means and standard deviations are reported for continuous variables

### Event-level associations between specific motives and cannabis use outcomes (study aim 1)

At the event-level, coping, social, enhancement, and expansion motives were associated with more sessions of cannabis use, a longer duration of intoxication, and higher subjective intoxication above and beyond other motives (Table 3). Sleep motives were associated with more sessions and higher subjective intoxication, and medical motives were associated with a longer duration of intoxication. Coping, expansion, and conformity motives were also associated with more consequences of use.

**Table 3.** Within-person associations between specific motives and cannabis use outcomes

Variable	Number of Sessions			Duration Intoxication			Subjective Intoxication			Consequences		
	*b*	*95% CI*	*p*	*b*	*95% CI*	*p*	*b*	*95% CI*	*p*	*b*	*95% CI*	*p*
Coping	0.15	0.05, 0.25	0.004	0.39	0.26, 0.53	< 0.001	0.14	0.08, 0.21	< 0.001	0.09	0.01, 0.18	0.04
Social	0.40	0.27, 0.52	< 0.001	0.53	0.37, 0.70	< 0.001	0.18	0.10, 0.27	< 0.001	− 0.01	− 0.11, 0.09	0.91
Enhancement	0.20	0.11, 0.29	< 0.001	0.57	0.46, 0.69	< 0.001	0.29	0.24, 0.35	< 0.001	0.05	− 0.02, 0.12	0.15
Medical	0.11	− 0.01, 0.23	0.08	0.27	0.08, 0.48	0.01	0.07	− 0.01, 0.15	0.08	0.11	− 0.01, 0.24	0.09
Sleep	0.13	0.02, 0.25	0.02	0.09	− 0.05, 0.23	0.22	0.07	0.004, 0.14	0.04	0.03	− 0.05, 0.11	0.44
Expansion	0.15	0.003, 0.29	0.04	0.36	0.18, 0.55	< 0.001	0.16	0.06, 0.25	< 0.001	0.24	0.11, 0.27	< 0.001
Conformity	0.05	− 0.11, 0.21	0.56	0.13	− 0.12, 0.37	0.30	0.09	− 0.02, 0.20	0.11	0.20	0.04, 0.36	0.01

All motives were entered as simultaneous predictors in a single model. Covariates included: within-person (day of EMA period, weekend vs. weekday, and morning vs. evening survey); between-person (race/ethnicity, sexual identity, gender identity, age)

Associations among variables at the between-person level were also estimated but are not included in tables

### Event-level associations with number of motives (study aim 2)

At the event-level, endorsing more motives for cannabis use was associated with more sessions of cannabis use, being intoxicated for longer, becoming more intoxicated, and experiencing more consequences during the same observation compared to when fewer motives were endorsed (Table 4). Observations during which some specific motives were endorsed were associated with endorsing additional motives. Endorsing conformity motives for cannabis use was associated with endorsing more additional motives for use during the same observation, while endorsing coping and sleep motives were associated with endorsing fewer additional motives. Social, enhancement, medical, and expansion motives were not associated with reporting additional motives at the event-level.

**Table 4.** Within-person associations with number of motives

Variable	*b*	*95% CI*	*p*
Number of Sessions	0.21	0.15, 0.26	< 0.001
Duration Intoxication	0.37	0.31, 0.43	< 0.001
Subjective Intoxication	0.17	0.14, 0.20	< 0.001
Consequences	0.10	0.06, 0.14	< 0.001
Specific Motives			
Coping	− 0.24	− 0.31, − 0.17	< 0.001
Social	0.05	− 0.05, 0.15	0.32
Enhancement	− 0.01	− 0.08, 0.06	0.76
Sleep	− 0.22	− 0.31, − 0.12	< 0.001
Medical	0.02	− 0.10, 0.13	0.78
Expansion	0.09	− 0.03, 0.22	0.16
Conformity	0.19	0.02, 0.35	0.02

Covariates included: within-person (day of EMA period, weekend vs. weekday, and morning vs. evening survey); between-person (race/ethnicity, sexual identity, gender identity, age)

Associations among variables at the between-person level were also estimated but are not included in tables

### Multilevel latent class analysis: identifying the number of classes (study aim 3)

We conducted a multilevel latent class analysis of observations during which multiple motives for cannabis use were reported to determine which motives were most likely to co-occur and if these different sets of motives were differentially associated with cannabis use outcomes. As analyses indicated that expansion and conformity motives did not help to differentiate classes, coping, social, enhancement, sleep, and medical motives were included as indicators of between 2 and 8 latent classes at the event-level. Model fit indices largely supported a seven-class solution as indicated by the lowest AIC, BIC, and sample-size adjusted BIC values and significance of the BLRT (Table 5). The VLMR suggested a preference for a six-class solution, and the seven-class solution included two relatively small classes (50 and 66 observations) that approached the lower limit for the proportion of the sample in a class (~ 1%). Therefore, we examined six- and seven-class solutions to determine which produced the most interpretable latent classes. Five of the latent classes were equivalent across the six- and seven-class solutions. In the six-class solution, a *social and enhancement* class was present that was no longer present in the seven-class solution. In the seven-class solution, two new classes emerged: *sleep and medical* and *coping*,* enhancement*,* and sleep*. Given the small sizes of the new classes that emerged in the seven-class solution and their lower theoretical interest compared to the *social and enhancement* class that was present in the six-class solution, we decided upon the six-class solution.

**Table 5.** Fit indices for latent class analysis of multiple motive observations

Number of Classes	AIC	BIC	Sample Size Adjusted BIC	Entropy	VLMR and BLRT Estimate	VLMR *p* value	BLRT *p* value	Smallest Class Size
2	15121.89	15187.80	15152.85	0.64	1054.68	< 0.001	< 0.001	1337
3	14625.83	14727.68	14673.67	0.88	508.06	< 0.001	< 0.001	719
4	14182.48	14320.28	14247.20	0.98	455.35	< 0.001	< 0.001	563
5	14105.02	14278.76	14186.62	0.91	89.46	0.03	< 0.001	310
6	14044.64	14254.33	14143.12	0.91	72.32	**0.01**	< 0.001	129
7	**14003.55**	**14249.19**	**14118.92**	0.93	56.83	0.06	**< 0.001**	50
8	14022.05	14303.63	14154.30	0.86	0.08	1.00	1.00	0

Lower BIC and adjusted BIC values indicate a better fitting model. Significant VLMR and BLRT indicate a preference for the current model over the model with one less class

*BIC* Bayesian information criterion, *VLMR* Vuong Lo-Mendell-Rubin likelihood ratio test, *BLRT* Bootstrapped likelihood ratio test

Bold text indicates the preferred model for each statistic

The six-class solution included the following classes (Table 6), each of which were characterized by 100% probability of endorsing each of the motives included in the class name unless otherwise specified: (1) *coping and enhancement* [*n* = 970]; (2) *coping and medical* [*n* = 516]; (3) *coping*,* enhancement*,* and medical* [*n* = 310; probability of endorsing coping motives was 85%]; (4) *coping*,* social*,* and enhancement* [*n* = 402; probability of endorsing enhancement motives was 69%]; (5) *social and enhancement* [*n* = 129]; and (6) *coping and sleep* [*n* = 628; probability of endorsing coping motives was 89%].

**Table 6.** Proportion of motives endorsement for each class

Class Number	Class Name	*n*	Coping Motives	Social Motives	Enhancement Motives	Sleep Motives	Medical Motives
Class 1	Coping and Enhancement	970	**1.00**	0.03	**1.00**	0.09	0.01
Class 2	Coping and Medical	516	**1.00**	0.02	0.13	0.00	**1.00**
Class 3	Coping, Enhancement, and Medical	310	**0.85**	0.21	**1.00**	0.29	**1.00**
Class 4	Coping, Social, and Enhancement	402	**1.00**	**1.00**	**0.69**	0.07	0.00
Class 5	Social and Enhancement	129	0.02	**1.00**	**1.00**	0.03	0.03
Class 6	Coping and Sleep	628	**0.89**	0.03	0.24	**1.00**	0.43

Proportion of cannabis use observations during which each motive was endorsed

Proportions that distinguish each class are bolded

### Differences across classes in cannabis use outcomes (study aim 3)

Follow-up multilevel analyses indicated several differences in cannabis use outcomes across latent classes (Table 7). During observations when *coping*,* social*,* and enhancement* motives were endorsed, participants reported more sessions of cannabis use compared to all other multiple motive classes; reported a longer duration of intoxication compared to when *coping and medical*,* coping and sleep*, or *coping and enhancement* motives were endorsed; became more intoxicated compared to when *coping and medical* or *coping and sleep* motives were endorsed; and reported more consequences than when *coping and medical* motives were endorsed. The other class characterized by endorsement of three motives (*coping*,* enhancement*,* and medical*) was also associated with more sessions of cannabis use, longer duration of intoxication, heavier intoxication, and more consequences compared to observations when *coping and medical* motives were endorsed as well as with heavier intoxication compared to observations when *coping and sleep* motives were endorsed.

**Table 7.** Mean differences across latent classes

Class	Number of Sessions		Duration of Intoxication		Subjective Intoxication		Consequences	
	*M*	*SE*	*M*	*SE*	*M*	*SE*	*M*	*SE*
Coping, Social, and Enhancement	1.68^a^	0.81	3.87^a^	0.81	3.07^a^	0.31	1.68^a^	0.40
Coping and Medical	1.11^b^	0.81	3.12^b^	0.81	2.79^b^	0.31	1.54^b^	0.40
Coping and Sleep	1.20^b, c^	0.81	3.47^c^	0.81	2.85^b^	0.31	1.55^a, b^	0.40
Coping and Enhancement	1.21^b, c^	0.81	3.54^c^	0.81	2.99^a^	0.31	1.54^a, b^	0.40
Coping, Enhancement, and Medical	1.41^c^	0.81	3.73^a, c^	0.81	3.01^a^	0.31	1.74^a^	0.40
Social and Enhancement	1.25^b, c^	0.81	3.53^a, c^	0.81	2.99^a, b^	0.31	1.49^a, b^	0.41

Superscript letters represent the results follow-up mean comparisons with corrections for multiple comparisons

Means with superscript letters that differ from one another indicate that the means differed significantly from one another

Some differences across classes characterized by endorsement of two motives were also present, largely differences between *coping and medical* motives and other classes. When *coping and medical* motives were endorsed, participants were intoxicated for a shorter period compared to observations when *coping and sleep*, *coping and enhancement*, or *social and enhancement* motives were endorsed. Further, when *coping and medical* or *coping and sleep* motives were endorsed, participants reported becoming less intoxicated than observations when *coping and enhancement* motives were endorsed.

## Discussion

The current study aimed to expand our understanding of event-level effects of motives for cannabis use utilizing three approaches: unique effects of each motive, count of the number of motives endorsed, and latent class analysis of specific combinations of motives. Results highlighted the utility of each of these analytic approaches. Examining the unique effects of each motive demonstrated several differences in cannabis use outcomes but results could quantify the effect of endorsing multiple motives. Endorsing more motives (using a count of motives) was associated with heavier cannabis use and more consequences, and utilizing this technique allowed us to identify motives that were more likely to co-occur with additional motives. However, this analysis could not identify differences based on the combination of motives endorsed. A latent class analysis of motives at the event-level identified a diverse set of motive combinations that tended to be endorsed together. Further, cannabis use outcomes differed based not only on the number of motives endorsed but also on the specific combination of motives endorsed. These findings indicate that important nuance is lost when number of motives is operationalized as a count.

Utilizing the most common analytic approach for examining associations with motives, we entered all motives as simultaneous predictors of cannabis use outcomes at the event-level to identify each motive’s unique effect. Results indicated that all motives, except conformity, were associated with heavier cannabis use based on at least one variable (i.e., number of sessions, duration of intoxication, subjective intoxication), but only a subset were associated with more consequences (i.e., coping, expansion, and conformity). Within-person associations between coping, social, and enhancement motives and more sessions, longer duration of intoxication, and higher subjective intoxication supported our hypotheses. While we hypothesized that coping, social, and enhancement motives would all be associated with experiencing more consequences, coping motives (but not social or enhancement motives) were associated with more consequences. These findings were largely consistent with the preponderance of evidence for event-level associations with coping, enhancement, and social motives, although the existing literature is somewhat mixed (Bonar et al. 2017; Dyar and Feinstein 2025; Jackson et al. 2021; Pearson et al. 2020). Given limited research on conformity, expansion, sleep, and medical motives at the daily level, we did not have hypotheses regarding the effects of these motives on cannabis use outcomes. These findings expanded the literature on motives that have been examined by fewer studies, including conformity, expansion, sleep, and medical. In short, this approach is useful for understanding how each specific motive is associated with cannabis use outcomes above and beyond any other motives that may be endorsed during the same observation. However, this approach produced similar patterns of results for many motives, which did not help to differentiate their effects. This pattern, with most motives being associated with heavier use, is also suggestive of an additive effect of motives because endorsing more motives would be associated with heavier use (via the addition of multiple motive effects). This approach also controls for the use of multiple motives, by including all as predictors in the same model, but cannot determine the effect of endorsing multiple motives or specific combinations of motives.

Building upon the results of Stevens et al. (2021), we next examined the event-level associations between a count of the number of motives endorsed and cannabis use outcomes. Consistent with Stevens et al. (2021) and our hypotheses, we found that endorsing more motives was associated with more sessions and consequences of cannabis use. Further, we added to this literature by demonstrating that endorsing multiple motives was also associated with longer intoxication and higher subjective intoxication. We also found that some motives were differentially associated with endorsing additional motives during the same observation. Specifically, conformity motives were associated with endorsing additional motives, while coping and sleep were associated with endorsing fewer additional motives. These results highlight the importance of attending to the number of motives endorsed, given that endorsing more motives was associated with heavier use and more consequences. However, this approach is not able to differentiate between observations in which the same number of motives were endorsed but the specific motives differed.

Therefore, we identified sets of motives that tended to co-occur by conducting a multilevel latent class analysis of motives during observations when more than one cannabis use motive was endorsed. This analysis identified six combinations of motives that were most commonly reported together during the same observation, most of which included coping motives and one or two additional motives. Only one class did not include coping motives – the *social and enhancement* motive class. The preponderance of coping motives across classes is consistent with the prevalence of coping motives in the full set of observations as coping motives were endorsed on 79% of observations during which cannabis use was reported. Medical and sleep motives, which are rarely examined at the event-level, characterized several classes (e.g., *coping and sleep*; *coping and medical*; *coping*,* enhancement*,* and medical*), highlighting the importance of assessing these motives at the event-level in future research.

Several differences emerged across these multiple motive classes. Overall, observations characterized by endorsement of three motives tended to be associated with heavier use than observations characterized by the endorsement of two motives, which is consistent with the results of analyses using a count of motives. However, results were not consistent across all two and three motive classes. Observations when *coping*,* social*,* and enhancement* motives were endorsed were consistently characterized by more sessions and longer intoxication compared to observations in which two motives were endorsed. However, this pattern was not as consistent for observations when *coping*,* enhancement*,* and medical* motives were endorsed. Specifically, observations during which *coping*,* enhancement*,* and medical* motives were endorsed only had more sessions and longer intoxication compared to observations when *coping and medical* motives were endorsed, while observations during which *coping*,* social*,* and enhancement* motives were endorsed were associated with more sessions and longer intoxication compared to all or most of the classes characterized by endorsement of two motives.

Notable differences also emerged among classes characterized by the endorsement of two motives, with observations during which *coping and medical* motives were endorsed being associated with lighter use than most other classes characterized by endorsement of two motives. Further, both observations when *coping and medical* and *coping and sleep* motives were endorsed were associated with lower subjective intoxication compared to observations when *coping and enhancement* motives were endorsed. Overall, these results highlight several differences among observations characterized by endorsement of two or three motives based on the combination of motives endorsed. These differences are obscured when a count of the number of motives is used.

### Clinical and research implications

Findings have several potential implications for clinicians working with clients who use cannabis regularly. Endorsing more motives on the same day was associated with heavier use and more consequences, so clinicians may ask clients to consider using more protective behavioral strategies on days when they are using cannabis for multiple reasons. Given that days when coping, social, and enhancement motives were endorsed were associated with particularly heavy cannabis use, this may be one type of multiple motive day that participants may be cautioned against or may call for the utilization of additional protective behavioral strategies. Endorsing both coping and medical or coping and sleep motives were associated with lighter use than many other combinations of motives, suggesting that days characterized by this combination of motives may not require additional protective behavioral strategies. Clinicians may also work with clients to identify particular combinations of motives that are associated with heavier use or more consequences for their clients and develop plans for reducing consequences on days when these higher risk sets of motives are endorsed.

This study also has implications for future research on motives for cannabis use at the daily level. Our results indicate that multiple motives are endorsed on many cannabis use days, highlighting the importance of considering motives for cannabis use together rather than examining the unique effects of specific motives. Further, our findings indicate that some combinations of motives are associated with heavier use than others, above and beyond the effect of simply endorsing multiple motives. This also suggests that additional insights may be gained by research that considers the unique effects of specific combinations of motives compared to studies that examine the unique effects of each motive or a count of motives. Research examining potential differences in contexts, modes, and timing of different multiple motive classes may identify potential mechanisms that may also help to explain the unique effects of specific combinations of motives. Future research that examines participants’ subjective experiences with particular combinations of motives via qualitative interviewing may help to further our understanding of why particular motive combinations are associated with heavier or lighter cannabis use.

### Limitations

The current study should be considered in light of its limitations. Given that the sample was comprised of 18–25 year old females who used cannabis regularly and lived in the US, it is unclear whether findings will generalize to males, other age groups, lower frequencies of cannabis use, or other countries. The sample also included an oversampling of sexual minority women and gender diverse individuals assigned female at birth. As there were too few heterosexual women to provide ample power to conduct sensitivity analyses of the latent class analyses with this subset of the sample, it is unclear whether the same set of classes would be identified in a sample exclusively comprised of heterosexual women. Sexual minority women and gender diverse individuals experience additional stressors as a result of the stigmatization of sexual and gender minorities (Meyer 2003) and utilize cannabis at higher rates than heterosexual women (Krueger et al. 2020; Schuler and Collins 2020). It has been theorized that these added stressors and more permissive norms about cannabis use in the sexual and gender minority community lead to differences in motive endorsement for sexual and gender minorities compared to heterosexual cisgender individuals (Hatzenbuehler, 2009). Further, these unique risk factors may also lead to different impacts of motives for sexual minority women and gender diverse individuals compared to heterosexual women (Hatzenbuehler, 2009). This sample also only included females, and some studies have suggested that there may be sex differences in motive endorsement and the impact of cannabis use motives for males and females in samples from the general population (e.g., Gex et al. 2023; Kroon et al. 2023; Morris and Buckner 2023). Therefore, future research should test whether the same classes of multiple motive endorsement identified in the current study generalize to other samples or whether they differ based on sex, gender, and sexual minority status.

Second, motives were assessed using binary items, rather than the more commonly used Likert response scales. While this reduced participant burden, this approach may have lost some nuance that may have been present if a Likert scale was used. Third, we were unable to take uncertainty in class assignment into account in analyses comparing latent classes to one another due to the lack of an analytic platform that could implement a more appropriate two or three step approach for the multilevel latent class solution identified in this manuscript. This concern is somewhat alleviated by the high entropy of the final latent class solution (0.91), but it would be more ideal to account for this uncertainty. Fourth, we did not define the bounds of a cannabis use session or occasion for participants. Although this is consistent with the approach used in the most common measure of cannabis use patterns (Cuttler and Spradlin 2017), participants may have varied in how they responded to this question. However, the focus of analyses on within-person associations controls for any individual-level differences in how participants operationalized a session or occasion. Therefore, within-person associations with number of sessions of cannabis use per occasion examined deviations from an individuals’ typical number of sessions, mitigating any potential individual-level differences in how participants defined cannabis use sessions.

## Conclusions

Results of the current study advance our understanding of associations between motives and cannabis use outcomes at the event-level, particularly during observations when multiple motives are endorsed. Our findings replicated the results of Stevens et al. (2021) by demonstrating that endorsing multiple motives for cannabis use was associated with heavier cannabis use and more consequences and built upon this prior study by identifying specific motives that were more likely to co-occur with additional motives. We then explored whether specific combinations of motives were differentially associated with cannabis use outcomes above and beyond the number of motives endorsed. Results indicated differences among motive classes with the same number of motives. For example, the two classes characterized by the endorsement of three motives displayed different patterns with one (*coping*,* social*,* and enhancement*) being consistently associated with heavier use than most classes characterized by endorsing two motives, while the other (*coping*,* enhancement*,* and medical*) was not consistently associated with heavier use. Further, one of the classes characterized by endorsement of two motives (*coping and medical*) was associated with lighter use compared to the other classes characterized by the endorsement of two motives. This highlights the nuances that are gained by examining specific combinations of motives rather than using a count of the number of motives.

## Data Availability

Data and materials are available from the authors upon request.

## Footnotes

1 Cannabis use criteria were selected to have adequate power (which increases as the number of cannabis use days reported increases) while maintaining broader generalizability (by keeping the criteria for the minimum number of cannabis use days reported at baseline low) and to be broadly consistent with inclusion criteria of other EMA studies of cannabis use.

## References

[CR1] Amiet D, Youssef GJ, Hagg LJ, Lorenzetti V, Parkes L, Solowij N, Yücel M. Young adults with higher motives and expectancies of regular cannabis use show poorer psychosocial functioning. Front Psychiatry. 2020;11:599365. 33384630 10.3389/fpsyt.2020.599365 PMC7771276

[CR2] Asparouhov T, Muthén B. (2010). Bayesian analysis of latent variable models using Mplus. Mplus Technical Reports. https://www.statmodel.com/download/BayesAdvantages18.pdf

[CR3] Asparouhov T, Muthén B. Auxiliary variables in mixture modeling: using the BCH method in Mplus to estimate a distal outcome model and an arbitrary secondary model. Mplus Web Notes. 2014;21(2):1–22.

[CR4] Bakk Z, Kuha J. Two-step estimation of models between latent classes and external variables. Psychometrika. 2018;83(4):871–92. 29150817 10.1007/s11336-017-9592-7

[CR5] Bonar EE, Goldstick JE, Collins RL, Cranford JA, Cunningham RM, Chermack ST, Blow FC, Walton MA. Daily associations between cannabis motives and consumption in emerging adults. Drug Alcohol Depend. 2017;178:136–42. 28647681 10.1016/j.drugalcdep.2017.05.006 PMC5548614

[CR6] Cloutier RM, Kearns NT, Knapp AA, Contractor AA, Blumenthal H. Heterogeneous patterns of marijuana use motives using latent profile analysis. Subst Use Misuse. 2019;54(9):1485–98. 31017512 10.1080/10826084.2019.1588325 PMC6833943

[CR7] Cuttler C, Spradlin A. Measuring cannabis consumption: psychometric properties of the daily sessions, frequency, age of onset, and quantity of cannabis use inventory (DFAQ-CU). PLoS One. 2017. 10.1371/journal.pone.0178194. 28552942 10.1371/journal.pone.0178194 PMC5446174

[CR8] Depaoli S, Clifton JP. A bayesian approach to multilevel structural equation modeling with continuous and dichotomous outcomes. Struct Equation Modeling: Multidisciplinary J. 2015;22(3):327–51.

[CR9] Dyar C, Feinstein BA. Event-level contextual and motivational risk factors for cannabis use: evidence for differing associations based on individual-level patterns of cannabis use among sexual minority women and gender diverse individuals. Psychol Sex Orientat Gend Divers. 2025;12(1):86–98. 10.1037/sgd0000645. 40092970 10.1037/sgd0000645 PMC11906180

[CR10] Dyar C, Kaysen D, Newcomb ME, Mustanski B. Event-level associations among minority stress, coping motives, and substance use among sexual minority women and gender diverse individuals. Addict Behav. 2022. 10.1016/j.addbeh.2022.107397. 35700652 10.1016/j.addbeh.2022.107397 PMC9732144

[CR11] Dziak JJ, Lanza ST, Tan X. Effect size, statistical power and sample size requirements for the bootstrap likelihood ratio test in latent class analysis. Struct Equ Model. 2014;21(4):534–52. 10.1080/10705511.2014.919819. 10.1080/10705511.2014.919819 PMC4196274 25328371

[CR12] Ferreira J, Zwinderman A. On the benjamini–hochberg method. Ann Stat. 2006;34(4):1827–49.

[CR13] Gex KS, Gray KM, Davis CN, Squeglia LM, McRae-Clark AL, Saladin ME, Tomko RL. Sex differences in the relationship between cannabis use motives and cannabis craving in daily life in emerging adults. Psychol Addict Behav. 2023;37(6):809. 37471012 10.1037/adb0000945 PMC10528470

[CR14] Goodhines PA, Gellis LA, Ansell EB, Park A. Cannabis and alcohol use for sleep aid: a daily diary investigation. Health Psychol. 2019;38(11):1036. 31169378 10.1037/hea0000765 PMC6800769

[CR15] Hatzenbuehler ML. How does sexual minority stigma “get under the skin”? A psychological mediation framework. Psychological Bu lletin. 2009;135(5):707. 10.1037/a0016441. 10.1037/a0016441 PMC2789474 19702379

[CR16] Hipp JR, Bauer DJ. Local solutions in the estimation of growth mixture models. Psychol Methods. 2006;11(1):36. 16594766 10.1037/1082-989X.11.1.36

[CR17] Jackson KM, Stevens AK, Sokolovsky AW, Hayes KL, White HR. Real-world simultaneous alcohol and cannabis use: an ecological study of situational motives and social and physical contexts. Psychol Addict Behav. 2021;35(6):698–711. 10.1037/adb0000765. 34472880 10.1037/adb0000765 PMC8487914

[CR18] Kroon E, Mansueto A, Kuhns L, Filbey F, Wiers R, Cousijn J. Gender differences in cannabis use disorder symptoms: a network analysis. Drug Alcohol Depend. 2023;243:109733. 10.1016/j.drugalcdep.2022.109733. 36565568 10.1016/j.drugalcdep.2022.109733

[CR19] Krueger EA, Fish JN, Upchurch DM. Sexual orientation disparities in substance use: investigating social stress mechanisms in a National sample. Am J Prev Med. 2020;58:59–68. 10.1016/j.amepre.2019.08.034. 31761516 10.1016/j.amepre.2019.08.034 PMC6925636

[CR20] Lee CM, Kilmer JR, Neighbors C, Cadigan JM, Fairlie AM, Patrick ME, Logan DE, Walter T, White HR. A marijuana consequences checklist for young adults with implications for brief motivational intervention research. Prev Sci. 2021;22(6):758–68. 33098002 10.1007/s11121-020-01171-x PMC8088772

[CR21] Lüdtke O, Marsh HW, Robitzsch A, Trautwein U, Asparouhov T, Muthén B. The multilevel latent covariate model: a new, more reliable approach to group-level effects in contextual studies. Psychol Methods. 2008;13(3):203. 18778152 10.1037/a0012869

[CR22] Meyer IH. Prejudice, social stress, and mental health in lesbian, gay, and bisexual populations: conceptual issues and research evidence. Psychol Bull. 2003;129(5):674–97. 10.1037/0033-2909.129.5.674. 12956539 10.1037/0033-2909.129.5.674 PMC2072932

[CR23] Morris PE, Buckner JD. Cannabis-related problems and social anxiety: the roles of sex and cannabis use motives updated. Addict Behav. 2023;137:107528. 10.1016/j.addbeh.2022.107528. 36335786 10.1016/j.addbeh.2022.107528

[CR24] Muthén B. (2010). Bayesian analysis in Mplus: A brief introduction. Mplus Technical Report.

[CR25] Nylund KL, Asparouhov T, Muthén BO. Deciding on the number of classes in latent class analysis and growth mixture modeling: a Monte Carlo simulation study. Struct Equ Model. 2007;14(4):535–69.

[CR26] Patrick ME, Fairlie AM, Cadigan JM, Abdallah DA, Larimer ME, Lee CM. Daily motives for alcohol and marijuana use as predictors of simultaneous use among young adults. J Stud Alcohol Drug. 2019;80:454–61. 10.15288/jsad.2019.80.454 PMC6739644 31495383

[CR27] Pearson MR, Bravo AJ, Conner BT, Parnes JE. A day in the life: a daily diary examination of marijuana motives and protective behavioral strategies among college student marijuana users. J Drug Issues. 2020;50(2):142–56. 10.1177/0022042619890837 PMC13557193 42719834

[CR28] Schuler MS, Collins RL. Sexual minority substance use disparities: bisexual women at elevated risk relative to other sexual minority groups. Drug Alcohol Depend. 2020. 10.1016/j.drugalcdep.2019.107755. 31810051 10.1016/j.drugalcdep.2019.107755 PMC6980764

[CR29] Simons JS, Dvorak RD, Merrill JE, Read JP. Dimensions and severity of marijuana consequences: development and validation of the marijuana consequences questionnaire (MACQ). Addict Behav. 2012;37(5):613–21. 22305645 10.1016/j.addbeh.2012.01.008 PMC3307958

[CR30] Stevens AK, Drohan MM, Boyle HK, White HR, Jackson KM. More reasons, more use and problems? Examining the influence of number of motives on consumption and consequences across alcohol-only, cannabis-only, and simultaneous-use days. J Stud Alcohol Drug. 2021;82(6):782–91. 10.15288/jsad.2021.82.782 PMC8819619 34762038

[CR31] Watson RJ, Fish JN, McKay T, Allen SH, Eaton L, Puhl RM. Substance use among a National sample of sexual and gender minority adolescents: intersections of sex assigned at birth and gender identity. LGBT Health. 2020;7(1):37–46. 31755811 10.1089/lgbt.2019.0066 PMC6983732

